# Information and communication integration in smart factory design

**DOI:** 10.12688/f1000research.122355.1

**Published:** 2022-09-09

**Authors:** Christian Fauska, Jaroslava Kniežová

**Affiliations:** 1 Faculty of Management, Comenius University in Bratislava, Bratislava, Bratislava, Slovakia

**Keywords:** Industry 4.0, smart factory design, shop floor management, information and communication technology

## Abstract

Strategic smart factory design is essential to utilize Industry 4.0 technologies in production environments effectively. Although a series of earlier reviews in the context of smart manufacturing have been published, so far none addresses smart factory design, i. e. the planning and operation of smart factories. This review provides an overview of recent research in the field by systematizing opportunities, risks and success factors of smart factory design as available from recent empirical studies (2018-2022). Businesses are informed how smart factory design should be approached and implemented to realize cost advantages and increase competitiveness. Academic research benefits of a classification of relevant issues and open research fields are outlined.

## Introduction

Smart factory design has become a buzzword in production engineering in the context of the Industry 4.0 debate: According to a study by PWC (PricewaterhouseCoopers) consultants, 91% of industrial companies are investing in digital factory technology in Europe and 90% of survey participants believe that opportunities of smart factory design outweigh its risks from a business perspective ( PWC, 2022).

Digital data processing enables the automation of all production steps and their and autonomous flow integration ( Mabkhot *et al*., 2018; Rejikumar *et al*., 2019). Intelligent value chains and product life cycles supporting the way of products from design to recycling have been called “Industry 4.0 technologies”. Industry 4.0 is characterized by on-time interoperability of virtual decentral and service oriented modular systems in the supply chain ( Rejikumar *et al*., 2019). This extends to the usage phase of smart products, *i.e.* intelligent and networked products indicating producers the necessary life cycle data and retrieving operation data automatically through the Internet of Things ( Brettel *et al*., 2014).

Smart factories comprise self-organizing virtually interlinked autonomous supply chain production and delivery environments which integrate production, machinery, and information technology based on decentral information and communication infrastructures ( Rejikumar *et al*., 2019). These cyber-physical systems self-organize essential production flows but also provide interlinks for human intervention and human integration in the work process, which is essential for control and supervision. Social machines collaborate with human beings by retrieving or analyzing provided data and images according to man-defined requirements or supporting human activities physically or informationally ( Zavadska & Zavadsky, 2018).

The integration of information and communication in smart factory environments offers important opportunities to realize economies of scale, save resources and use manpower more efficiently ( Oesterreich & Teuteberg, 2016). To implement smart factory designs at the informational level, however, huge amounts of data have to be collected stored, retrieved and analyzed to develop and supervise production routines and order flows autonomously. This puts high requirements on information technology technology development and maintenance.

Smart factory design refers to factory layout, planning and construction aimed to integrate machines and production based on Industry 4.0 information technology ( Kagermann *et al*., 2013). Machinery and equipment communicate *via* the Internet of Things a virtual networking platform ( Osterrieder *et al.*, 2020). Based on that technology, smart factories could span the whole supply and delivery chain: social machines, *i.e.* communicative self-organized IT technology could communicate across factories and digital production technologies. Different companies could interact locally and self-reliantly ( Mabkhot *et al*., 2018).

This study evaluates opportunities, challenges and success factors of information and communication in smart factory design based on a systematic literature review of empirical studies to outline the status of existing literature, identify further empirical research fields and inform companies how to make smart factory environments succeed.

## Earlier related reviews

Although a series of topical (2018 and beyond) reviews in the context of Industry 4.0 and smart manufacturing are available, the research field of information and communication integration in smart factory design has not yet been explored: Rejikumar *et al*. (2019) retrieve distinguishing attributes of Industry 4.0 applications from earlier studies and analyze the content and timeline of publications, but do not explicitly discuss opportunities and challenges of the technologies. Fatorachian and Kazemi (2020) evaluate impacts of Industry 4.0 on supply chain performance, a part sector of smart manufacturing. Erro-Garcés (2019) conducts a meta-analysis of Industry 4.0 studies published between 2005 and 2018 and highlights managerial issues. With its Industry 4.0 focus, this approach is broader than the current study, does not explicitly mention challenges, and does not include the most recent papers. Calabrese *et al*. (2020) conduct a review of opportunities, difficulties and development goals of Industry 4.0 technologies and systematize nine technologies, among them smart worker and smart equipment technologies which are part of the smart manufacturing process, but does not conclusively describe them. The study lacks an analysis of success factors.

Five recent reviews in the context of smart manufacturing are available: Yang *et al*. (2018) systematize recent research trends in smart manufacturing based on a review. The review by Osterrieder *et al*. (2020) of smart factory studies systematizes technical solutions and identifies groups of technologies to outline a digital value stream. Hughes *et al*. (2020) discuss future potentials of Industry 4.0 applications to manufacturing but do not assess presently available technologies. None of these studies discusses smart factory design, its potentials, risks or success factors.

Some specialized reviews partly address potentials and risks of smart manufacturing in certain contexts: Mabkhot *et al*. (2018) identify “perspectives of smart factory applications and technical support systems for smart factory implementation” in the form of proprieties of smart production and smart products but does not refer to smart factory planning and construction. The study also neglects potential risks of smart manufacturing. Lee *et al*. (2018) discuss literature in machine health management ( *i.e.* maintenance and repair) in smart factories, but do not refer to smart factory design. Mittal *et al*. (2019) critically review the applicability of available smart manufacturing and Industry 4.0 maturity models to SME and diagnose low adaptiveness to small business manufacturing practices. All three studies focus on a part segment of smart manufacturing and are thus narrower in range than the intended study and problem rather than solution focused.

So far, no review systematically juxtaposes the opportunities and risks of information and communication integration in smart factory design based on a comprehensive analysis of presently available technologies and referring to the most recent studies. This review aims to close this research void.

## Methods

This study conducts a systematic review based on a methodology suggested by Synder (2019). The purpose of the study is to identify and analyze topical empirical research in information and communication integration in smart factory design. Three key research questions are meant to be answered:

• What are opportunities of smart factory applications from the perspective of production company applications?
• Which problems of smart factory applications have been observed?
• What can be done to design smart factory applications so that opportunities are fulfilled while problems are avoided?

**Figure 1.**
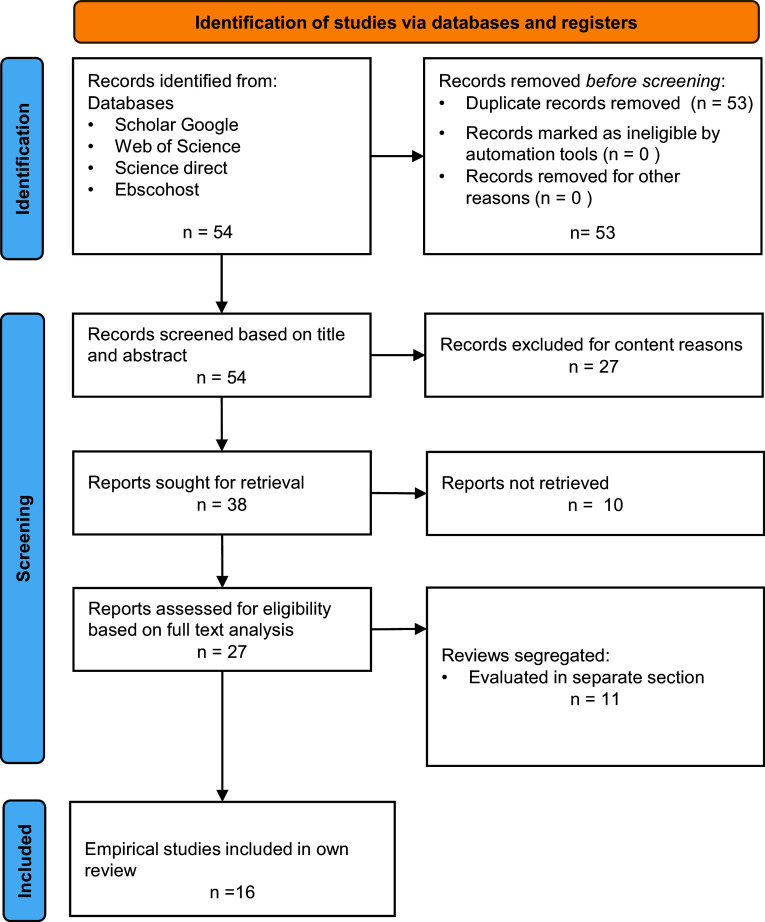
Outline of review process.

Figure 1 outlines the literature research process: To identify eligible studies the review uses a systematic research strategy. The review is limited to studies in English published in peer reviewed journals or at academic conferences in the period 2018 to 2022. To systematize the research process, the databases EbscoHost, Web of Knowledge, Science Direct and Scholar Google were consulted using the following homogenous keyword combination: [“smart factory design” OR “smart factory] AND [information OR communication] AND [review OR empirical]” The databases sort by relevance and studies are considered until a point of saturation is reached *i.e.* no further eligible studies are found or results repeated.

A secondary manual evaluation process deselects reviews and then discards non-empirical studies, studies of minor empirical quality and studies that do not fit content-wise, *i.e.* do not focus on smart factory applications but are more general, *e.g.* on Industry 4.0. From the initially identified 54 studies (based on the key word combination), 11 are identified as reviews (see above) and deselected from the core analysis, 27 are discarded due to lacking focus on “smart manufacturing” or lacking empirical evidence, 16 remain for the final review. For a graphical overview of the literature research process (see Figure 1).

First the studies are summarized in an author-centric table addressing sample, research method, identified opportunities, limitations and success factors and points of critique. This step corresponds to Webster & Watson’s (2002) content matrix. The study to catalogue the studies and extract relevant major and subcategories, a so-called concept matrix is drafted, which reorganizes the points made by arguments in major and sub-items and structures the textual evaluation (compare appendix). The textual evaluation of the studies follows the organization of the concept tables.

Based on a synthesis of the review results, opportunities and risks of smart factory technologies are juxtaposed. Drawing on the outlined success factors the potentials to resolve inherent risks are discussed. Further research requirements are given if adequate solutions to recognized risks of smart factory design are unavailable.

## Results: I&C integration in smart factory design

The appendix provides an overview of the retrieved studies in the form of a content matrix ( Table 1). Further classification is seen in concept matrices of opportunities, risks and success factors of smart factory design ( Table 2, Table 3 and Table 4 respectively) ( Webster & Watson, 2002). The arguments for opportunities, risks and success factors are each classified into technical informational, economic and sustainability (social or environmental) aspects. Technical aspects dominate the discussion and refer to the planning phase and the operation phase of smart factories. This structure guides the following sections:

**Table 1.** Overview on reviewed studies.

Empirical research in smart manufacturing – 2018 to 2022					
Study	Industry/application	Potentials	limitations	Success factors	Point of critique
Baek, 2021	Adaption and monitoring of vibration sensor signals in automated storage	Real time fault analysis Digital failure detection	High function complexity	Digital failure management Facilitate automatic prognosis	Limited risk assessment
Braccini & Margherita, 2018	Sustainability of smart manufacturing, case study	Improved productivity & product quality Continuity of energy consumption Safer work environment, lower human work load Job enrichment			No critical reflections
Büchi *et al*., 2020	Analysis of success factors of Industry 4.0 application in manufacturing units		High investment costs Risk of obsolescence	Openness to technical innovation Depth, breadth of Industry 4.0 technology Modularity of Industry 4.0 local units	
Guo *et al*., 2019	Usage of digital twin to model smart factory design	Quick analysis of design options Early identification of design errors Time saving Reduce costs early in planning phase	Complexity at planning stage	High real-world fidelity of digital twin Accurate parameter validation Control strategies Virtual model correspondence to physical data structures	Study limited to design stage
Häckel *et al*., 2019	Analysis of IT security risks in smart factory networks using graph theory and value at risk	Real time information synchronization Automated communication	IT security risks IT component non-availability Complex network structures High investment Rapid development cycles	Tight IT security measures Appropriate support required Access accuracy and accountability solutions	

1 ^st^ Author	Industry/application	Potentials	limitations	Success factors	Point of critique
Jin & Lee, 2018	Smart factory construction Korean metal working firms		Complex planning process	CEOs progressive intentions Innovative lending 6 investment Framing industrial characteristics Firm growth and high R&D activity	Limited focus on I&C technology
Ko *et al.*, 2020	113 smart factories in Korea	Improved final product quality Process optimization	Securing and managing facility data Product demand forecast Low job creation	Integrate modular systems Targeted yield management Supervising defect rate	Limited to (early) Korean smart factories
Lee, 2021	Survey among workers in smart factories in Korean SME	Production line supervision Improved efficiency & productivity Flexible production	High investment costs Limited supply chain abilities	ERP system Targetted quality management Ethical management: CEO support Productivity management systems Work process standardization	Limited to Korea (only partly comparable to more industrialized countries)
Li *et al*., 2019	Embedding big data solutions in smart factories Semistructured interviews			Top management commitment Business-IT strategic alignment Appropriate hardware (cloud, communication technology, big data) Supply chain integration	
Mantravadi *et al*., 2022	Industrial Internet of Things in manufacturing Case study	Interoperability Quality improvement Responsiveness Flexibility to different IoT devices		Develop standardized interfaces Ensure modularity Use open-source components	Single case only University industrial lab
Micheler *et al*., 2019	Smart technologies in Industry 4.0 Online survey & workshop	Higher sustainability Informational networking Self-organizing shop-floors	Resource constraints Connection between modular systems Dynamic & extreme environmental conditions disturb connectivity Compatibility problems, complexity Lacking strategic guidance	Ensure system compatibility particularly hard & software Utilization of cloud technologies	
Suebsook *et al*., 2020	Smart manufacturing in Thailand	Increase firm performance *i.e.* market share, cost reduction by interoperability technological advances and service orientation			
Schaupp & Diab, 2020	Interviews with smart factory managers in Germany	Automate the self-organization of industrial capital	Shift of personal control from management to cybernetic control Replacement of direct labour by autonomous systems		Pure economic viewpoint, no success factors
Vestin *et al*., 2018	Smart factories for wood house builders Single case study	Improved competitiveness due to economies of scale	Maturity of culture & organization to modern work methods		
Wang & Lee, 2021	5G communications in smart factory scenarios		Limited available measurement data	Indoor path loss prediction analysis Training of path loss models based on neural networks	
Xia *et al*., 2021	Digital twin system for smart factory construction application in hydraulic cylinder production	Reduction of work-in process inventory Save delivery time Virtual reality simulation, mapping & control		Definition of digital twin on basis of product lifecycle requirements Refinement of digital twin in planning &construction phase Reliance on Building information management database Analyze user requirements	

### Opportunities of smart factory design

Only two studies ( Guo et al., 2019; Xia et al., 2021) refer to technical opportunities in the planning stage of smart factories, *i.e.* the actual design phase, and suggest to develop and refer to a digital twin of the planned factory to simulate the production environment first ( Guo *et al*., 2019). Digital twins are electronic usually 3-dimensional models which are developed and refined in the planning process. They comprise building-related information, machinery equipment data and are extended to simulate production flows and interconnections in the supply chain ( Xia *et al*., 2021). Digital twins allow the flexible analysis of design options and realistic simulation of production conditions. Simulations in the planning stage avoid erroneous designs and avoid ill-designed physical plants ( Guo *et al*., 2019; Xia *et al*., 2021).

Most evaluated contributions however assess the technical advantages of smart factories in operation as compared to conventional production ( Baek, 2021; Ko *et al*., 2020; Micheler *et al*., 2019; Braccini & Margherita, 2018; Mantravadi *et al*., 2020; Lee, 2021; Suebsook *et al*., 2020). The transition to smart manufacturing by designing a smart factory offers diverse advantages for businesses:

Smart factories contribute to an optimization of production process flows ( Ko *et al*., 2020) and partly enable fully self-organizing shop floors ( Micheler *et al*., 2019), which saves manpower on the shop floor and frees human resources for responsible control and supervision tasks. Production in automated supply and processing chains is adapted to demand at short notice, *i.e.* is on-time responsive to order flows ( Lee, 2021).

Smart manufacturing realizes product quality improvements due to high automation quotas and digital control and planning solutions ( Braccini & Margherita, 2018; Ko *et al*., 2020) Smart factories usually dispose of real time digital failure analysis, which facilitates error detection and avoidance ( Baek, 2021). Human workers are discharged of responsibility.

The advantages of smart factory design at the informational level refer to the informational model backing technical implementation at the manufacturing machines and in the logistics of the production process. Digital media synchronize information flows across workshops, storages and machines on time ( Häckel *et al*., 2019). Machines interact and communicate in a self-organized manner without necessary human intervention ( Schaupp & Diab, 2020). Standardized production processes are run through the value chain automatically ( Häckel *et al*., 2019). An extensive informational network systematizes the production process based on earlier flow data ( Micheler *et al*., 2019).

Friction less order flows presuppose the interoperability of the IT systems of production machines and IT manufacturing planning systems ( Mantravadi *et al*., 2022; Suebsook *et al*., 2020). Information and communication architectures are designed flexible to adapt to different Internet of Things, devices which allows a flexible composition of the production chain ( Mantravadi *et al*., 2022). Work process inventories can be reduced on that basis ( Xia *et al*., 2021) and manpower is saved for responsible extraordinary information management tasks ( Micheler *et al*., 2019).

Technical and informational opportunities of smart factory design produce economic advantages. As compared to conventional production smart manufacturing sites frequently realize productivity increases ( Braccini & Margherita, 2018; Lee, 2021). Demand based production reduces redundancies and allows efficiency gains ( Lee, 2021).

Smart manufacturing saves time in the inner organizational order flow ( Guo *et al*., 2019) and equally reduces delivery time due to just-in-time planning ( Xia *et al*., 2021). Realized economies of scale reduce costs and increase business competitiveness ( Vestin *et al*., 2018).

Social and environmental sustainability of smart manufacturing sites can be increased as compared to conventional production ( Micheler *et al*., 2019) due to higher energy consumption continuity ( Braccini & Margherita, 2018). Information technology-supported production machines are worker friendly and service oriented, which improves work conditions and satisfaction on the job ( Suebsook *et al*., 2020).

**Table 2.** Concept matrix of opportunities of smart factory design.

Opportunities	subcategory	source
Technical in planning	Digital twin: design options analysis Virtual reality simulation, mapping & control	Guo *et al*., 2019 Xia *et al*., 2021
Technical in operation	Real time, digital fault analysis Error avoidance	Baek, 2021
	Process optimization	Ko *et al*., 2020
	Self-organizing shop floors	Micheler *et al*., 2019
	Product quality improvement Improved final product quality Quality improvement	Braccini & Margherita, 2018 Ko *et al*., 2020 Mantravadi *et al*., 2022
	Production flexibility, responsibleness	Lee, 2021
	Technological advance	Suebsook *et al*., 2020
Informational	Real time information synchronization	Häckel *et al*., 2019
	Automated communication Self-organization	Häckel *et al*., 2019 Schaupp & Diab, 2020
	Informational networking	Micheler *et al*., 2019
	Cost reduction by digital twin in planning phase	Guo *et al*., 2019
	interoperability	Mantravadi *et al.*, 2022 Suebsook *et al*., 2020
	Flexibility to different IoT devices	Mantravadi *et al*., 2022
	Reduced work in process inventories	Xia *et al*., 2021
Economic	Productivity increase	Braccini & Margherita, 2018
	Improved efficiency & productivity	Lee, 2021
	Time saving Reduced delivery time	Guo *et al*., 2019 Xia *et al*., 2021
	Increased firm performance	Suebsook *et al*., 2020
	Economies of scale - competitiveness	Vestin *et al*., 2018
Sustainability/social	Energy consumption continuity	Braccini & Margherita, 2018
	Higher sustainability	Micheler *et al*., 2019
	Service orientation	Suebsook *et al*., 2020

### Risks of smart factory design

Technical risks of smart factory design at the planning stage are often concerned with the excessive complexity of site and equipment layout ( Jin & Lee, 2018). Guo *et al*. (2019) are concerned about the potentially lacking adequacy or over-sophistication of the “digital twin” *i.e.* building information management model, which impairs its operability and puts the reproducibility of simulation results at risk. Due to rapid planning cycles and dynamic technological development ( Häckel *et al*., 2019) smart manufacturing equipment is threatened by obsolescence ( Büchi *et al*., 2020). Häckel *et al*. (2019) fear compatibility problems among IT and production machines and incompatibility between the diverse modular units of the plant. Limited availability of measurement data could impair the prognosis and early identification of compatibility issues ( Wang & Lee, 2021).

At the stage of operation, technical problems could emerge due to high function complexity ( Häckel *et al*., 2019), which entails a high number of interactions between modular production devices and the corporate enterprise resource planning architecture ( Baek, 2021). Incorrect demand forecasts resulting from technical malalignment could mean a major threat to the implementation of efficient smart manufacturing systems ( Ko *et al*., 2020). Häckel *et al*. (2019) fear lacking operationality of smart manufacturing equipment due to the failure and temporary unavailability of essential components. The limited operability of individual Industry 4.0 components could endanger the flow of the whole production process if all units are interdependent and automatized ( Micheler *et al*., 2019).

Informational risks of smart factory design are frequently connected to IT security ( Häckel *et al*., 2019). Autonomous and interdependent systems and complex network architectures relying on the web 2.0 as a communication channel have been exposed to hacker attacks and data abuse ( Ko *et al*., 2020). In smart manufacturing, complex network architectures intermesh the whole supply chain. Limited capabilities of supply chain partners ( Lee, 2021), can impair the functioning of the whole logistic process and make high sophisticated solution at the core company redundant ( Häckel *et al*., 2019). Studies further discuss low strategic guidance and orientation from inhouse management with regard to the conclusive implementation of smart factory designs ( Micheler *et al*., 2019). Leaders feel a loss of personal control if information and manufacturing technologies interact self-reliantly and are reluctant to admit further digitalization steps ( Schaupp & Diab, 2020). According to Vestin *et al*. (2018), lacking organizational adaptiveness to modern technologies is a major reason for the failure or inadequate implementation of smart factory technologies.

Economic restrictions to smart factory design are repeatedly mentioned in the retrieved studies ( Büchi *et al*., 2020; Häckel *et al*., 2019; Lee, 2021). Companies fear that the investment in smart factory architectures will not amortize due to lower-than-expected efficiency gains ( Häckel *et al*., 2019). High investment costs in digital solutions are a major reason to stick to established analogous production systems. Businesses facing resource constraints are partly unable to gain investment partners for innovative Industry 4.0 solutions if the profitability is uncertain ( Micheler *et al*., 2019).

Finally, smart factory design is assumed to be little responsible from a social perspective: Smart manufacturing hardly creates new jobs but makes workers with low qualification redundant ( Ko *et al*., 2020). Human labor is replaced by a network of self-reliant machines and information and communication technology ( Schaupp & Diab, 2020).

**Table 3.** Concept matrix of risks of smart factory design.

Risks	subcategory	source
Technical in planning stage	Complexity at planning stage	Guo *et al*., 2019 Jin & Lee, 2018
	Risk of obsolescence	Büchi *et al*., 2020
	Rapid planning cycles	Häckel *et al*., 2019
	Compatibility problems	
	Limited measurement data	Wang & Lee, 2021
Technical in operation stage	High function complexity	Baek, 2021 Häckel *et al*., 2019
	Component non-availability	Häckel *et al*., 2019
	Correct demand forecast	Ko et al., 2020
	Unconditional operability	Micheler *et al*., 2019
Informational	IT security risks	Häckel *et al*., 2019
	Securing and managing data	Ko *et al*., 2020
	Complex network structures	Häckel *et al*., 2019
	Limited supply chain capabilities	Lee, 2021
	Modular system interconnections	Micheler *et al*., 2019
	Low strategic guidance/orientation	Micheler *et al*., 2019
	Loss of personal control	Schaupp & Diab, 2020
	Organizational adaptiveness to modern technologies	Vestin *et al*., 2018
Economic	High investment costs	Büchi *et al*., 2020 Häckel *et al*., 2019 Lee, 2021
	Resource constraints	Micheler *et al*., 2019
Sustainable/social	Low job creation	Ko *et al*., 2020
	Replacement of human labour	Schaupp & Diab, 2020

### Success factors of smart factory design

Success factors of smart factory design targeted at controlling technical, informational and economic risks and caveats. At the technical planning stage of smart factories, the real-world fidelity of the digital factory model (digital twin) is essential. It is gradually adapted to real data structures and production flows as planning progresses ( Guo *et al*., 2019). Xia *et al*. (2021) explained that the development of a digital twin requires a detailed analysis of the product life cycle and extensive data bases of the building information model, which is continuously updated and fed with the most recent production data.

Modular systems are resilient to disruptions in the value chain or temporary information lacks since they can accomplish their tasks self-reliantly, even if part of the production network breaks down ( Ko *et al*., 2020). On the other hand, strict modularity based on common technical standards is essential to fit the value creation chain together and interconnect it in virtual space ( Büchi *et al*., 2020). Mantravadi *et al*. (2022) recommended the application of standardized modular interfaces to ensure adaptiveness when the line’s process flow has to be changed or new equipment is integrated. Hardware and software, *e.g.* the digital databases, should be fully integrated ( Li *et al*., 2019; Mantravadi *et al*., 2020), which requires electronic system compatibility across all levels of the value chain ( Micheler *et al*., 2019). Büchi *et al*. (2020) advise that planning adequate breath, and depth of Industry 4.0 technology is essential to ensure sustainable evolution of the smart factory when novel technologies emerge in future or a redesign of the production process is required.

In technical operation, smart factories should be equipped with a detailed productivity management system to direct order flows through the system effectively. Baek (2021) emphasizes the relevance of reliable automated prognostic tools to schedule production planning based on a data base ( Guo *et al*., 2019). To keep automated production systems running, accurate parameter validation and control is indispensable which again is based on a gapless information management system ( Ko *et al*., 2020). To ensure high production quality of automated manufacturing systems these should dispose of an equally digitalized quality management concept and rely on standardized work processes as much as possible ( Lee, 2021). The detailed supervision of defect rates through that system allows to recognize deviances early and human intervention should be possible without delay in that case ( Ko *et al*., 2020).

The informational basis is key to operate smart factories without friction, which comprises an effective failure management ( Baek, 2021). Wang & Lee (2021) exemplify this by a digital path loss training algorithm based on 5G technology which intervenes in case of erroneous production flows. Maximum IT security standards are required to keep self-reliant smart manufacturing systems safe and running. Access limitations and clear accountability regulations are fundamental to the informational safety of the production line. This includes adequate (human) IT support in case of extraordinary events ( Häckel *et al*., 2019). As Li *et al*. (2019) observe, smart factory effectiveness and sustainability depend on a conclusive strategic business-IT alignment scheme, which includes supply chain interaction. Micheler *et al*. (2019) suggest relying on cloud technologies for the storage and sharing of huge data volumes in that inter-business network.

To make smart factory design an economic success, businesses should dispose of the necessary managerial and cultural preconditions: Business culture should be open to innovation ( Büchi *et al*., 2021), which as Jin & Lee (2018) explain depends on the progressive attitude of the top management. Leaders should be involved and committed to Industry 4.0 technologies to guide businesses on the long way to autonomous production and accept the necessary investments in sustainable technology ( Li *et al*., 2019). An environment of high research and development activity and strongly growing companies is advantageous to the frictionless implementation of smart production systems since companies usually have to rely on innovative lending and investment partners to implement their strategy. A stringent yield management is essential to monitor the efficiency of smart production sites ( Ko *et al*., 2020).

**Table 4.** Concept matrix of success factors of smart factory design.

Success factors	subcategory	source
Technical in planning	Real world fidelity of digital twin Virtual model correspondence to physical data structures	Guo *et al*., 2019
	Analysis of product livecycle requirements (digital twin) Reliance on BMI data	Xia *et al*., 2021
	Modularity of technology Modular systems Modular standardized interfaces	Büchi *et al*., 2020 Ko *et al*., 2020 Mantravadi *et al*., 2022
	Appropriate hardware & data integration System compatibility	Li *et al*., 2019 Mantravadi *et al*., 2020 Micheler *et al*., 2019
	Breadth & depth of Industry 4.0 technology	Büchi *et al*., 2020
Technical in operation	Reliable automated prognostic tools	Baek, 2021
	Accurate parameter validation and control	Guo *et al*., 2019
	Supervision of defect rates	Ko *et al*., 2020
	Productivity management system	Ko *et al*., 2020
	Work process standardization	
	Effective quality management	Lee, 2021
Informational	Effective failure management	Baek, 2021
	Path loss training in production flows	Wang & Lee, 2021
	Tight IT security	Häckel *et al.*, 2019
	Adequate IT support	Häckel *et al*., 2019
	Access limitation and accountability	Häckel *et al*., 2019
	Strategic business-IT alignment	Li *et al.*, 2019
	Supply chain integration	Li *et al*., 2019
	Cloud technology usage	Micheler *et al*., 2019
Economic	Openness to technical innovation Progressive CEO intentions Top management commitment	Büchi *et al*., 2021 Jin & Lee, 2018 Li *et al*., 2019
	High R&D activity, firm growth	Jin & Lee, 2018
	Innovative lending & investment	Jin & Lee, 2018
	Targeted yield management	Ko *et al*., 2020

## Discussion

### Implications for practitioners

Summarizing the review results, smart factory design opportunities, risks, and success factors are conclusively classified into five corresponding categories technical aspects in planning and operation, informational aspects, economic aspects and social/ecological aspects. Businesses benefit of some fundamental advice as to the planning and operation of smart factories.

To utilize design opportunities in technical planning proactively, businesses are required to control technical complexity at the planning stage, avoid compatibility issues and risks of rapid obsolescence. Digital twin simulation are useful to predict future physical performance and ensure the friction less interaction of all plant components in a modular design.

In technical operation smart manufacturing technology excels due to real time digital fault analysis, self-organizing shop floor environments and can realize higher quality standards at improved flexibility than conventional technologies. These benefits are threatened by low operability due to high system complexity and interdependency. To avoid these difficulties businesses should standardize operation and quality management routines and establish interlinks for early human intervention in case of difficulties.

At the informational level, smart factory design allows the automation of communication *via* self-organizing informational networks. In a real world application, however, IT security risks threaten plant operation and private data could be abused. Businesses risk losing control of production processes, and intervening late in case of failure, which can result in the costly failure of the entire production line. Low in-house competency to monitor and repair the plant, makes businesses dependent on expensive external experts. Businesses can reduce this dependence by developing in-house knowledge on their IT system and by applying tight IT security standards.

At the economic level, smart factories promise productivity increases, higher quality standards and in effect improved competitiveness. The investment costs to build smart factories, however, are significant. To amortize these expenses, smart production lines should be designed flexible to adapt to different production jobs and volumes. Investment or financing partners should be provided a reliable calculation of expected benefits of the smart factory.

If planned to requirements, smart factories can save energy, however, threaten unqualified jobs which are substituted by automated processes. Businesses should plan digitalization and automation early to develop their work force so that responsible jobs in machine and computer operation can be taken over by long-standing employees, while job cuts are avoided.

### Implications for academia and call for further research

The evaluation of recent (published 2018 to 2022) studies in smart factory design has provided some general insights in the opportunities, risks and success factors of smart factory design from a business perspective. Essential categories for classifying these issues have been developed, which can be used as a foundation to further empirical research in smart factory design. The literature analysis has found 11 reviews and 16 empirical studies fitting with the research objective, which suggests that available research in Industry 4.0 and smart factory design tends to be theoretical and literature focussed. In available empirical research, practice applications are frequently based on single case studies *i.e.* smart factory applications in individual companies ( Braccini & Margherita, 2018; Lee, 2021; Mantravadi *et al*., 2022; Vestin *et al*., 2018; Xia *et al*., 2021), which impairs the representativeness of these studies. Empirical studies differ in focus and range: Some focus on particular technologies ( *e.g.* Wang & Lee, 2021: 5G communications; Guo *et al*., 2019: digital twin; Häckel *et al*., 2019: IT security risks; Baek, 2021: vibration sensor signals in automated storage). Their results apply to specific conditions but are not generally applicable to smart factory design in other contexts. Other studies are very broad in range ( *e.g.* Büchi *et al*., 2020; Industry 4.0 application in manufacturing units; Jin & Lee, 2018: Smart factory construction in Korea; Micheler *et al*., 2019: Smart technologies in Industry 4.0). The results of these studies are broadly applicable but little concrete concerning concrete smart factory implementations. Businesses planning smart factory solutions, thus obtain little valuable information from current academic research.

Further empirical research in smart factory design is required, to systematize available smart manufacturing technologies and empirically analyse implementations of smart factory solutions, ideally in the form of a comparative analysis including several businesses. the issues of smart supply chain integration and man–machine interaction planning have hardly been addressed in recent empirical studies and further research in these fields of smart factory design is desirable.

### Study limitations

This study has provided an overview of recent empirical and review-based publications in smart factory design, has derived advice for businesses investing in the field and has outlined further academic research requirements. However, the insights gained here are limited in range. Only 27 studies (11 reviews and 16 empirical studies) have been referred to due to limitations in range. Publications before 2018 have not been considered. The provided overview on smart factory design research thus is not comprehensive and the inclusion of further studies would be useful to obtain a representative overview of available smart factory technologies and their potential integration.

## Author contributions

Collection and analysis were performed by Christian Fauska. The first draft of the manuscript was written by Christian Fauska, and all authors commented on previous versions of the manuscript. All authors read and approved the final manuscript.

## Data availability

### Underlying data

All data are available as part of the article.

### Reporting guidelines

FIGSHARE: PRISMA checklist and flowchart for ‘Information and communication integration in smart factory design: a systematic review’, https://doi.org/10.6084/m9.figshare.20279223.v1 ( Fauska & Kniežová, 2022).

Data are available under the terms of the Creative Commons Attribution 4.0 International license (CC-BY 4.0).
