# Supplementary material for: Information and communication integration in smart factory design
Source: F1000Res. 2023 Jul 21;11:1026. Originally published 2022 Sep 9. [Version 2] doi: 10.12688/f1000research.122355.2 (PMC12627937; doi:10.12688/f1000research.122355.2)
Supplement: Peer review history

# Reviewer report: Reviewer response for version 2

**DOI:** 10.5256/f1000research.152180.r429548

**Published:** 2025-11-18

**Authors:** Natheer Almtireen

**Affiliations:** 1 German Jordanian University, Amman, Amman Governorate, Jordan

The article presents a systematic review on the integration of information and communication technologies in smart factory design, focusing on opportunities, risks, and success factors derived from empirical studies published between 2018 and 2022. The topic is timely and relevant, and the authors provide a structured synthesis supported by content and concept matrices. The findings are organized effectively across technical, informational, economic, and social dimensions.

 The rationale and objectives are clearly stated, and the review addresses a focused research gap. The methodology is generally sound, with appropriate use of databases, a defined search string, and structured synthesis techniques. The results are systematically presented, and the discussion offers actionable insights for both practitioners and researchers.

 While the study is well executed overall, minor revisions are recommended to enhance clarity and rigor. Specifically, the authors should briefly clarify their selection criteria and screening process, and refine some expressions for consistency and precision. Quantifying key findings where possible would also strengthen the presentation.

 The article is suitable for indexing following minor improvements **in methodological clarity and language refinement. The content is scientifically valid and contributes usefully to the literature on Industry 4.0 and smart factory design.

Are the rationale for, and objectives of, the Systematic Review clearly stated?

Yes

Is the statistical analysis and its interpretation appropriate?

Not applicable

If this is a Living Systematic Review, is the ‘living’ method appropriate and is the search schedule clearly defined and justified? (‘Living Systematic Review’ or a variation of this term should be included in the title.)

Not applicable

Are sufficient details of the methods and analysis provided to allow replication by others?

Partly

Are the conclusions drawn adequately supported by the results presented in the review?

Yes

Reviewer Expertise:

Mechatronics, Robotics, Industry 4.0

I confirm that I have read this submission and believe that I have an appropriate level of expertise to confirm that it is of an acceptable scientific standard.

# Reviewer report: Reviewer response for version 2

**DOI:** 10.5256/f1000research.152180.r421777

**Published:** 2025-10-15

**Authors:** Walter Cardoso Sátyro

**Affiliations:** 1 Nove de Julho University (UNINOVE),, Sao Paulo, Brazil

Thank you for the opportunity to cooperate to improving the manuscript entitled "Information and communication integration in smart factory design".

 The purpose of the study is to identify and analyze topical empirical research in information and communication integration in smart factory design

 The subject is relevant.

 Earlier related reviews. Considering that the review time was carried on from 2018 to 2022, I invite the authors to reconsider this phrase: "...So far, no review systematically..." Suggestion...Until 2022, no review systematically..."

 Methods. 1. Please inform why you used the term "review" in the search string and after, in the secondary selection you excluded review papers.

 2. Please close the apostrophe in the search string "..."keyword combination: [“smart factory design” OR

 “smart factory **<==**]

 3. The PRISMA flowchart diagram was used, but not informed in the text not referenced in  Fig. 1. Please correct it. (https://www.prisma-statement.org/prisma-2020-flow-diagram)

 4. In Fig. 1, the number of documents identified in each Databases must be informed between parentheses next to each database (Identification), and the total number, before excluding the duplicated, informed (not 54 that is the result of the total minus the duplicated).

 5. In the text, please inform that 10 documents were not possible to retrieve, and if possible, provide the reason(s).

 6.Please consult some published papers with the PRISMA flowchart, to refine yours. The numbers of documents found in each stage is not correct.

 Results: I&C integration in smart factory design. 

 1. "...Human workers are discharged of

 responsibility.." I invite the authors to give the reference for this affirmation, or to exclude it. Humans always will have the responsibility for machine, AI or any other technology they use, no matter what.

 2. "...Social and environmental sustainability of smart manufacturing sites can be increased as compared to conventional

 production (Micheler et al., 2019) due to higher energy consumption continuity...". Please provide sense to this affirmation.

 Discussion.

 1. Implications for practitioners. Please use some references to give basis to your affirmation. You can use some form the Results section.

 2. Implications for academia and call for further research

 "The evaluation of **recent **(published 2018 to 2022) studies...". Please exclude "recent".

 Study limitations. Please inform as a limitation the time range of the study, published from 2018 to 2022.

Are the rationale for, and objectives of, the Systematic Review clearly stated?

Yes

Is the statistical analysis and its interpretation appropriate?

Not applicable

If this is a Living Systematic Review, is the ‘living’ method appropriate and is the search schedule clearly defined and justified? (‘Living Systematic Review’ or a variation of this term should be included in the title.)

Not applicable

Are sufficient details of the methods and analysis provided to allow replication by others?

Partly

Are the conclusions drawn adequately supported by the results presented in the review?

Yes

Reviewer Expertise:

Operational management, Industrial engineering, Industry 4.0/5.0, AI

I confirm that I have read this submission and believe that I have an appropriate level of expertise to confirm that it is of an acceptable scientific standard, however I have significant reservations, as outlined above.

# Reviewer report: Reviewer response for version 2

**DOI:** 10.5256/f1000research.152180.r210593

**Published:** 2023-10-13

**Authors:** Athanasios Kalogeras

**Affiliations:** 1 Industrial Systems Institute, Patras, Greece

The paper presents a literature review related to ICT integration in the context of smart factory design. The methodology followed for the review is sound although the number of papers reviewed seems really low for the topic. The authors identify opportunities, risks, and success factors related to this domain. They engage into a discussion with reference to implications for practitioners and issues open for future research. The paper is interesting and its structure is adequate.

 Some challenges that appear in the field seem to not be touched by the review either in the presentation of the review findings or in the subsequent discussion:

- Operational technology and Information technology integration is an open challenge for the manufacturing environment;
- Cloud / fog / edge computing combination could provide solutions for a more efficient factory design;
- Legacy system integration remains an open issue and is also related with the overall cost minimization;
- Some liaison to Industry 5.0 and the synergetic environment between machines and humans would be beneficial to the paper;
- Design across different plants and companies is somehow touched but relevant content could be enhanced.

Language check should be performed. Examples of inappropriate language use:

- “and their and autonomous flow integration”;
- “Internet of Things a virtual networking platform”;
- “Friction less order flows”;
- “to adapt to different Internet of Things, devices”;
- “As compared to conventional production smart manufacturing sites frequently realize productivity increases”.

Are the rationale for, and objectives of, the Systematic Review clearly stated?

Yes

Is the statistical analysis and its interpretation appropriate?

Partly

Are sufficient details of the methods and analysis provided to allow replication by others?

Yes

Are the conclusions drawn adequately supported by the results presented in the review?

Yes

Reviewer Expertise:

Integration and interoperability in the manufacturing environment; cyberphysical systems; Industrial Internet of things; agile manufacturing; enterprise information systems

I confirm that I have read this submission and believe that I have an appropriate level of expertise to confirm that it is of an acceptable scientific standard, however I have significant reservations, as outlined above.

# Reviewer report: Reviewer response for version 1

**DOI:** 10.5256/f1000research.134334.r173434

**Published:** 2023-06-02

**Authors:** Varun Tripathi

**Affiliations:** 1 Accurate Institute of Management & Technology, Greater Noida, India

The authors reviewed recent research works in the context of smart manufacturing by systematizing opportunities, risks, and success factors of smart factory design as available from recent empirical studies. The manuscript's contents are well short of the standard for publication in a research journal. There are several things that could be improved in the current research work.

 The authors should include the following suggestions in their manuscript for the manuscript to be considered:          

1. In the abstract, the authors should clarify the present research work's objective and describe how the study could benefit researchers. The authors should also explain the statement given in the abstract "So far none addresses smart factory design, i.e. the planning and operation of smart factories". The abstract must be modified systematically.
2. There are several discrepancies in the introduction section, as follows:
  a) What is the Research objective?
  b) What are the needs and current scenario?
  c) Describe the backgrounds.
  d) Which types of problems have been faced by researchers in previous research works?
  e) What is the meaning of the statement given "So far, no review systematically juxtaposes the opportunities and risks of information and communication integration in smart factory design based on a comprehensive analysis of presently available technologies and referring to the most recent studies".
3. The authors must include the latest research works on smart manufacturing.
4. The authors must review the literature extensively to understand the present condition of smart factory design.
5. The research outcomes should be described.
6. Which data collection and analysis tools/methods have been used?
7. The authors should explain the reason for the focus on selecting research studies for 2018–2022.
8. According to the journal standards, the references cited need to be more systematic.
9. The authors must add the description of tables 2, 3, and 4.
10. How and why the opportunities, risks, and success factors are same?
11. The authors should define the results obtained by analyzing opportunities and risks in the discussion section. The authors should describe how the researchers could consider the different categories in smart factory design. Which types of factors and limitations should consider for achieving desired outcomes?

Are the rationale for, and objectives of, the Systematic Review clearly stated?

Partly

Is the statistical analysis and its interpretation appropriate?

Partly

Are sufficient details of the methods and analysis provided to allow replication by others?

Partly

Are the conclusions drawn adequately supported by the results presented in the review?

No

Reviewer Expertise:

NA

I confirm that I have read this submission and believe that I have an appropriate level of expertise to state that I do not consider it to be of an acceptable scientific standard, for reasons outlined above.

# Response

**Published:** 2023-06-26

**Authors:** Christian Fauska

**Affiliations:** Comenius University in Bratislava, Germany

Hello,

 Thank you very much for your feedback. I have tried to improve the points in a 2nd version. Some points were, in my opinion, already described. Therefore, a short statement if I have not implemented everything in the point. Also, the 2nd Reviewer had partly contrary views. Therefore I tried to mediate:

 1. In the abstract, the authors should clarify the present research work's objective and describe how the study could benefit researchers. The authors should also explain the statement given in the abstract "So far none addresses smart factory design, i.e. the planning and operation of smart factories". The abstract must be modified systematically.

 --> The abstract is based on the possibilities of the journal. It gives only a first overview. In my opinion, all the points raised will only be clarified with the scope of the introduction.

 2. There are several discrepancies in the introduction section, as follows:

 a) What is the Research objective?

 --> It's here in the Introduction section in the last paragraph

 b) What are the needs and current scenario?

 --> As described in the objective: "This study assesses opportunities, challenges, and success factors of information and communication in designing smart factories based on a systematic literature review of empirical studies to outline the state of the existing literature, identify further empirical research areas, and inform companies on how to successfully design smart factories."

 c) Describe the backgrounds.

 --> This issue is highlighted in a separate chapter, "Earlier related reviews."

 d) Which types of problems have been faced by researchers in previous research works?

 --> It's here in the Introduction section in the last paragraph where it belong

 e) What is the meaning of the statement given "So far, no review systematically juxtaposes the opportunities and risks of information and communication integration in smart factory design based on a comprehensive analysis of presently available technologies and referring to the most recent studies".

 --> The sentence is correct. Rewritten: “To date, there is no review work that systematically compares the opportunities and risks of information and communication integration in the design of smart factories based on a comprehensive analysis of the currently available technologies and with reference to the latest studies. This review aims to fill this research gap.”

 3. The authors must include the latest research works on smart manufacturing.

 --> The article was written in 2021 and beginning 2022. Published in August 22. It was actually. I will develop to the newest development in other articles.

 4. The authors must review the literature extensively to understand the present condition of smart factory design.

 -->The reviews were even summarized and compared in tabular form. For the sake of simplicity, I have placed the tables at the end of the article. This way, they are a supplement to the body text.

 5.The research outcomes should be described.

 -->I discuss the findings in the Results chapter and discuss them in detail in the Discussion chapter based on the tabular analyses of the studies.

 6. Which data collection and analysis tools/methods have been used?

 --> according to Snyder (2019), the method is shown in detail and also graphically in the methods chapter.

 7. The authors should explain the reason for the focus on selecting research studies for 2018–2022.

 --> the study was written in 2021 and 2022. This is the actual status of research. This u forced also in your point 3.

 8. According to the journal standards, the references cited need to be more systematic.

 --> I have provided detailed tabular lists of all results and their methodological evaluation. This is all that can be systematized. We use a Proven Methodology according to Snyder (2019) and already present the results in detail also in tabular form and derive the whole evaluation text in detail.

 9.  The authors must add the description of tables 2, 3, and 4.

 --> The tables are the summary of the chapter. U ca see it as underlining the content.

 10. How and why the opportunities, risks, and success factors are same?

 --> Because they are the key enabler for success.

 11. The authors should define the results obtained by analyzing opportunities and risks in the discussion section. The authors should describe how the researchers could consider the different categories in smart factory design. Which types of factors and limitations should consider for achieving desired outcomes?

 --> They are combined. U can not unique them. U have to understand the whole technical topic with the factettes-  opportunities, risks, and success factors – to achieve outcomes.

# Reviewer report: Reviewer response for version 1

**DOI:** 10.5256/f1000research.134334.r153717

**Published:** 2022-11-07

**Authors:** Deepika Pandita

**Affiliations:** 1 Business Management, Symbiosis International University, Pune, India

The article is surely a very interesting one, but the following aspects needs to be worked on by the authors:

 1. The abstract needs to be re-worked very explicitly stating the need and objective of the study. The current abstract talks about only the overview of smart factories.

 2. The literature review is very concise and needs to be elaborated.

 3. Which methods of LR is used in this study? Please mention that very clearly.

 4. Research Questions need to be mentioned very clearly and should be in sync with the research objectives.

 5. The findings are missing. The authors need to mention these as they have studied various literature review papers, so they should include how these studies help to discuss the findings.

Are the rationale for, and objectives of, the Systematic Review clearly stated?

Yes

Is the statistical analysis and its interpretation appropriate?

Partly

Are sufficient details of the methods and analysis provided to allow replication by others?

Yes

Are the conclusions drawn adequately supported by the results presented in the review?

Yes

Reviewer Expertise:

human resources, organizational behavior, entreprenership

I confirm that I have read this submission and believe that I have an appropriate level of expertise to confirm that it is of an acceptable scientific standard, however I have significant reservations, as outlined above.

# Response

**Published:** 2023-06-26

**Authors:** Christian Fauska

**Affiliations:** Comenius University in Bratislava, Germany

Hello,

 Thank you very much for your feedback. I have tried to improve the points in a 2nd version. Some points were, in my opinion, already described. Therefore, a short statement if I have not implemented everything in the point. also, the 2nd Reviwer had partly contrary views. There I tried to mediate:

 1. the summary must be revised by explicitly stating the need and the goal of the study. The current summary only gives an overview of smart factories.

 --> It is described in detail in the introduction. I would also not elaborate on the possibilities of the descent owed here.

 2. the literature review is very brief and needs to be elaborated.

 --> In principle you want a longer overview. I don't want that, because other articles investigate the topics and it is shown that the scientific contribution is finished for me. I will include wieter anlehnungen in other articles

 3. what methods of LR are used in this study? Please specify.

 --> According to Snyder (2019), the method is shown in detail and also graphically in the methods chapter.

 4. The research questions must be stated very clearly and should be consistent with the research objectives.

 --> They are listed under the methods as follows:

 - What are the opportunities of smart factory applications from the perspective of applications in manufacturing companies?

 - What problems have been observed in smart factory applications?

 - What can be done to design smart factory applications to take advantage of the opportunities and avoid the problems?

 5. there is a lack of insights. The authors need to mention these as they have studied various literature reviews, so they should state how these studies help to discuss the findings.

 -->I discuss the findings in the Results chapter and discuss them in detail in the Discussion chapter based on the tabular analyses of the studies.
